# Glutamine supplementation stimulates cell proliferation in skeletal muscle and cultivated myogenic cells of low birth weight piglets

**DOI:** 10.1038/s41598-021-92959-6

**Published:** 2021-06-28

**Authors:** Yaolu Zhao, Elke Albrecht, Katja Stange, Zeyang Li, Johannes Schregel, Miriama Sciascia, Cornelia C. Metges, Steffen Maak

**Affiliations:** 1 Leibniz Institute for Farm Animal Biology (FBN), Institute of Muscle Biology and Growth, 18196 Dummerstorf, Germany; 2 Leibniz Institute for Farm Animal Biology (FBN), Institute of Nutritional Physiology “Oskar Kellner”, 18196 Dummerstorf, Germany

**Keywords:** Animal physiology, Cell growth, Transcription, Muscle stem cells, Immunohistochemistry

## Abstract

Muscle growth of low birth weight (LBW) piglets may be improved with adapted nutrition. This study elucidated effects of glutamine (Gln) supplementation on the cellular muscle development of LBW and normal birth weight (NBW) piglets. Male piglets (n = 144) were either supplemented with 1 g Gln/kg body weight or an isonitrogeneous amount of alanine (Ala) between postnatal day 1 and 12 (dpn). Twelve piglets per group were slaughtered at 5, 12 and 26 dpn, one hour after injection with Bromodeoxyuridine (BrdU, 12 mg/kg). Muscle samples were collected and myogenic cells were isolated and cultivated. Expression of muscle growth related genes was quantified with qPCR. Proliferating, BrdU-positive cells in muscle sections were detected with immunohistochemistry indicating different cell types and decreasing proliferation with age. More proliferation was observed in muscle tissue of LBW-GLN than LBW-ALA piglets at 5 dpn, but there was no clear effect of supplementation on related gene expression. Cell culture experiments indicated that Gln could promote cell proliferation in a dose dependent manner, but expression of myogenesis regulatory genes was not altered. Overall, Gln supplementation stimulated cell proliferation in muscle tissue and in vitro in myogenic cell culture, whereas muscle growth regulatory genes were barely altered.

## Introduction

Low birth weight (LBW) piglets, often occurring in large litters, have a greater mortality rate, altered lipid deposition and retarded body growth in comparison with their normal birth weight (NBW) littermates^1, 2^. The delayed growth of LBW piglets is mainly due to a slower development of skeletal muscle as the myogenic activity is usually impaired in these animals during the fetal and early postnatal period^3, 4^. These disadvantages could cause long-term negative effects in LBW piglets^1^ and different adapted feeding strategies have been developed to compensate the growth delay and improve the body composition of LBW piglets^5–7^. Feeding with amino acids can stimulate protein synthesis in all tissues, especially in skeletal muscle as reviewed by Davis et al.^3^. This stimulation effect decreases with age^8^, thus it is reasonable to support muscle development with additional amino acids during the suckling period of piglets. In this project, we aimed to ameliorate the retarded growth of LBW piglets by oral glutamine (Gln) supplementation. Wu and Knabe^9^ reported that free Gln in porcine milk increased from 0.1 to 4 mM between day 1 and 28 of lactation indicating the potential importance of Gln for pig development in the early postnatal phase. Glutamine is regarded as the most abundant amino acid in the body, is mainly synthesized in skeletal muscle^10^, and muscle tissue is the most important site for Gln storage^11^. It promotes cell proliferation as a precursor for the synthesis of purine and pyrimidine nucleotides and provides energy, when metabolized to glutamate^12, 13^. Furthermore, Gln serves as a precursor for the synthesis of arginine, which is indispensable for the optimal growth of neonatal piglets^14^ and plays important roles in essential metabolic pathways^15^. However, LBW piglets might not receive enough Gln from milk to compensate the delayed body growth^15^. Our previous studies showed that Gln had the potential to increase muscle fiber size in piglets^16^, but the mechanism was not clear. We observed a temporarily increased intramuscular availability of free Gln that could have influenced cellular processes in skeletal muscle. Therefore, we complemented our investigations into the cellular development of the skeletal muscle of neonatal piglets with in vitro studies using a primary porcine myogenic cell culture model with Gln supplementation.

Protein deposition occurs when protein synthesis rate is greater than the protein degradation rate, which contributes to the postnatal muscle growth together with myonuclei accretion^3, 17–19^. In general, nuclei from myoblasts are not able to divide when myoblasts have been incorporated into myofibers^20^. Thus, during this process, muscle satellite cells (also named muscle stem cells) are the origin of the increasing number of myonuclei^3, 20, 21^, providing growth and regeneration of muscle cells^22, 23^. However, the proliferative satellite cells are most abundant in piglets during the first days of life, and then decrease with age^24^.

A number of key factors regulate muscle growth and regeneration. Paired box transcription factor 7 (*PAX7*) is always expressed in quiescent satellite cells^25^, regulating muscle growth in the early phase of cell proliferation^26^, and restricting the muscle stem cell differentiation^27^. Peroxisome proliferator-activated receptor gamma coactivator 1-alpha (*PPARGC1A*) is a possible regulator of satellite cell activation^19^. When satellite cells are activated, the myogenic regulatory factors (MRFs) including myoblast determination protein 1 (*MYOD*), myogenic factor 5 (*MYF5*), myogenic factor 6 (*MYF6* or *MRF4*) and myogenin (*MYOG*) start to modulate the muscle cell differentiation^28^. It was shown that *MYOD*, *MYF5* and *MRF4* are regulators for satellite cell differentiation to myocytes in vivo and for early differentiation in vitro^23, 29^, whilst *MYOG* is indispensable for terminal differentiation^29, 30^. In contrast, myostatin (*MSTN*) is a negative regulator of muscle growth during the whole life^31^. Altogether, these genes are suited as biological markers when investigating muscle cell development.

Since Doumit and Merkel^32^ first isolated myogenic satellite cells from pig in 1992, porcine satellite cell culture has been considered as an ideal model for studying muscle cell growth and development including proliferation and differentiation^33^. Furthermore, the successful transplantation of myofibers generated from cultured satellite cells into mouse muscle^34^ underlined the suitability of this in vitro system.

Our hypothesis was that Gln supplementation could improve muscle growth in early postnatal piglets by stimulation of satellite cell proliferation, their differentiation to myoblasts and fusion with myofibers. The objective of the present study was to evaluate the impact of glutamine through two approaches, one in vivo and one in vitro. Therefore, we quantified cell proliferation and expression of involved myogenic regulator genes in the skeletal muscle of piglets differing in birth weight (BiW) and in a porcine myogenic cell culture model under the influence of a supplementation with Gln.

## Results

### Detection of cell proliferation within *M. longissimus*

**Figure 1:**
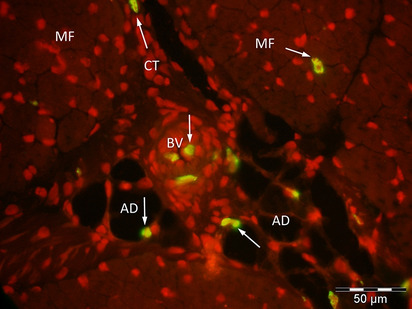
Incorporation of BrdU in proliferating cells within muscle tissue. Immunohistochemical detection of BrdU-positive nuclei (green) and total nuclei (red, stained with propidium iodide) in a longissimus muscle cross section of a 12-day-old piglet. Arrows indicate proliferating cells of different types. *MF* muscle fibers, *AD* adipocytes, *BV* blood vessel, *CT* connective tissue.

Incorporation of Bromodeoxyuridine (BrdU) into newly synthesized DNA was used to identify proliferating cells in muscle tissue. Proliferating, BrdU-positive cells in *Musculus longissimus dorsi* (MLD) were detected with immunohistochemistry (nuclei appear green in Fig. 1) close to muscle fibers (MF), but also within connective tissue (CT), near developing adipocytes (AD), and in blood vessels (BV). The localization suggests that different cell types were involved, such as satellite cells, fibroblasts, preadipocytes, endothelial cells, etc., but the individual cell types with BrdU-positive nuclei could not be quantified in this analysis.

**Figure 2:**
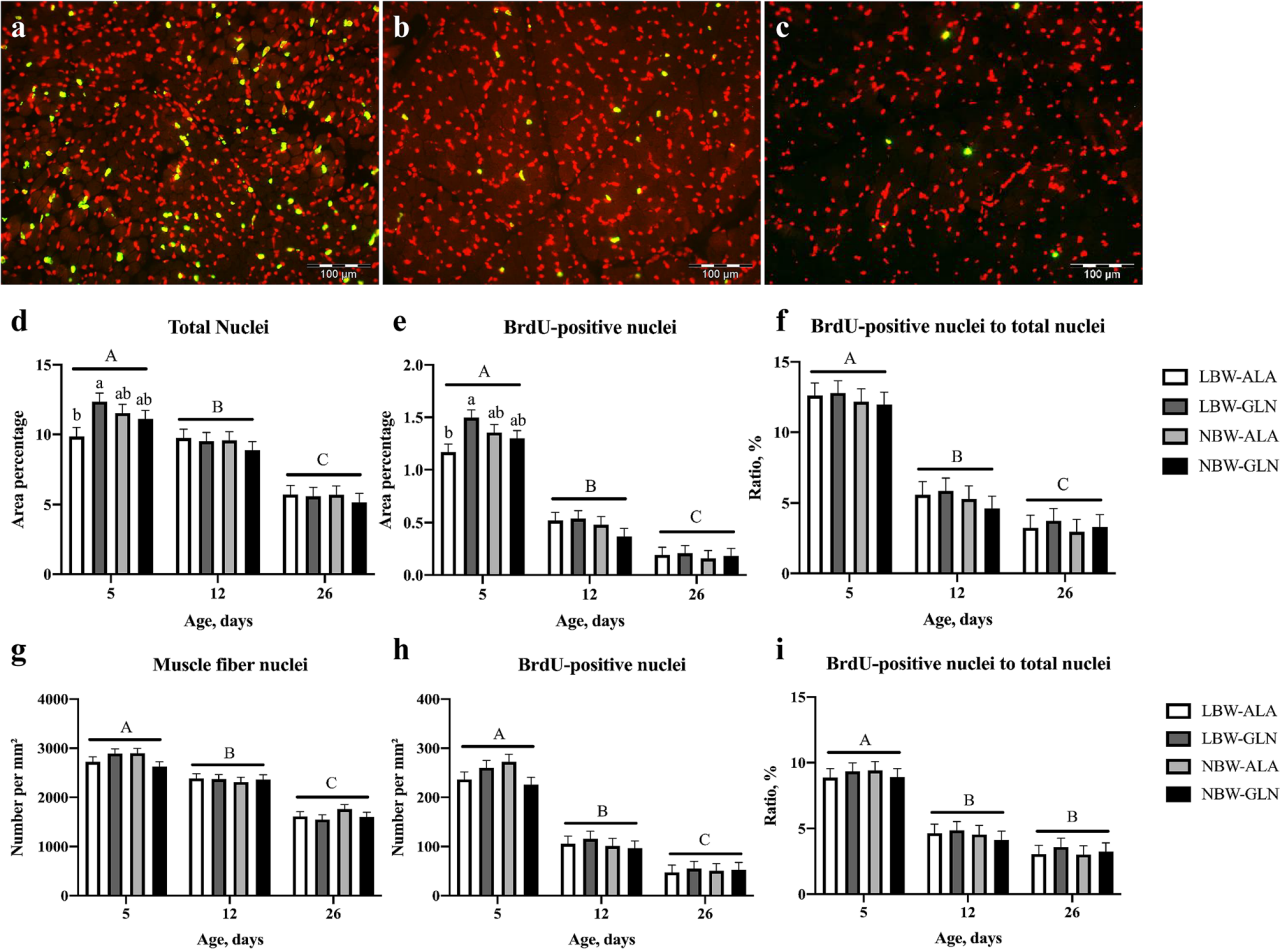
Detection and quantification of proliferating cells in *M. longissimus*. (**a**–**c**) Immunohistochemical detection of BrdU-positive nuclei (green) and total nuclei (red) at 5, 12 and 26 dpn; (**d**) area percentage of total nuclei; (**e**) area percentage of BrdU-positive nuclei; (**f**) ratio of BrdU-positive nuclei to total nuclei; (**g**) number of total nuclei per mm^2^ in a region comprising muscle fibers exclusively; (**h**) number of BrdU-positive nuclei per mm^2^; (**i**) ratio of BrdU-positive nuclei to total nuclei in *M. longissimus* of glutamine (GLN) or alanine (ALA) supplemented low birth weight (LBW) and normal birth weight (NBW) piglets at 5, 12 and 26 dpn. Values are presented as LSmeans and SE (n = 12 per group). Different uppercase letters (A-C) indicate significant differences among ages and different lowercase letters (a, b) indicate significant differences among groups within the same age (*p* ≤ 0.05, Tukey–Kramer test).

The number of BrdU-positive nuclei per area unit decreased with age (Fig. 2a–c). This observation was reflected by the area percentage of BrdU-positive nuclei that decreased from 5 days post-natum (dpn) to 26 dpn (*p* < 0.001, Fig. 2e). The total number of nuclei, measured as area percentage, decreased at the same time due to muscle fiber growth, but to a lesser extent (Fig. 2d). Thus, the ratio of BrdU-positive to total nuclei (Fig. 2f) also decreased from 5 to 26 dpn (*p* < 0.001). An influence of Gln supplementation was observed in LBW piglets at 5 dpn, indicating more total (*p* = 0.024) and more BrdU-positive nuclei (*p* = 0.009) in LBW-GLN than in LBW-ALA piglets. However, this difference was not observed in older piglets at 12 or 26 dpn (*p* > 0.1). The ratio of BrdU-positive nuclei to total nuclei was not influenced by BiW (*p* = 0.220) or Gln supplementation (*p* = 0.892, Fig. 2f). The numbers of total and BrdU-positive nuclei per mm^2^ in regions occupied exclusively by muscle fibers, without visible connective tissue, mainly represent satellite cell nuclei. Both were higher in piglets at 5 dpn than at 12 or 26 dpn (*p* < 0.001, Fig. 2g,h), without being influenced by BiW or Gln supplementation (*p* > 0.1). The ratio of BrdU-positive muscle nuclei to total muscle nuclei was not influenced by BiW (*p* = 0.583) or Gln supplementation (*p* = 0.820, Fig. 2i) as well.

### Expression of muscle growth-related genes in *M. longissimus*

**Figure 3:**
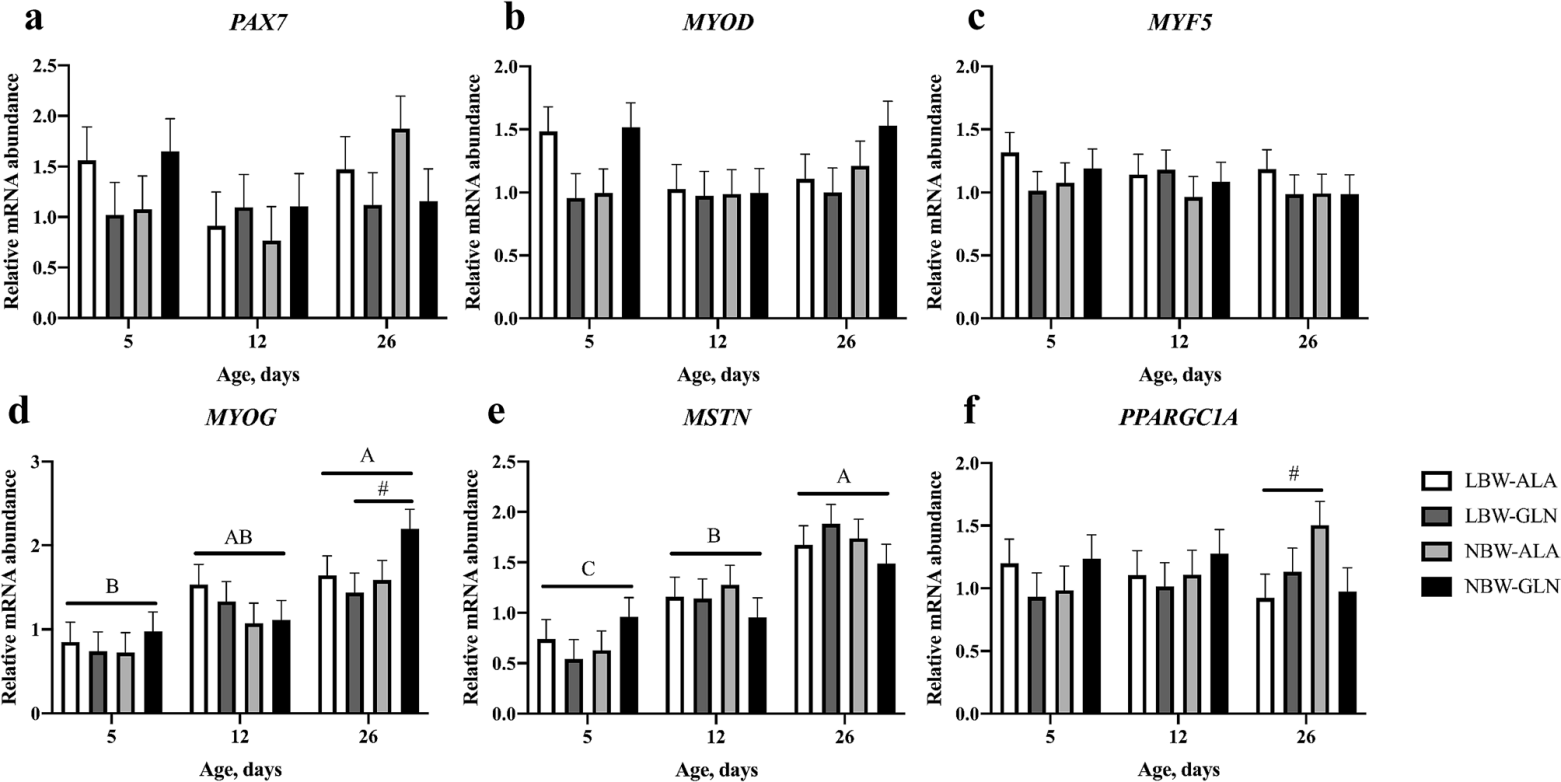
Relative mRNA abundances of genes in *M. longissimus*. Expressions of *PAX7* (**a**), *MYOD* (**b**), *MYF5* (**c**), *MYOG* (**d**), *MSTN* (**e**) and *PPARGC1A* (**f**) were analyzed in MLD of glutamine (GLN) or alanine (ALA) supplemented low birth weight (LBW) and normal birth weight (NBW) piglets at 5, 12 and 26 dpn (n = 12 per group). Values were normalized to *YHWAZ* and *PPIA* expression and are presented as LSmeans and SE. Different uppercase letters (A, B) indicate significant differences among ages (*p* ≤ 0.05, Tukey–Kramer test), ^#^trend among groups within the same age (0.05 < *p* ≤ 0.1).

The expression of muscle growth-related genes was quantified in MLD and is presented in Fig. 3. Relative mRNA abundances of *PAX7*, *MYOD* and *MYF5* were not affected by BiW, Gln supplementation or age (*p* > 0.1, Fig. 3a–c). The mRNA abundance of *MYOG* was higher in piglets at 26 dpn compared with animals at 5 dpn (*p* < 0.001, Fig. 3d). Moreover, *MYOG* tended to be less expressed in LBW-GLN than in NBW-GLN piglets (*p* = 0.067) at 26 dpn. The mRNA abundance of *MSTN* was greater in piglets at 26 dpn in comparison with piglets at 5 or 12 dpn (*p* < 0.05, Fig. 3e), but was not influenced by BiW or supplementation (*p* > 0.1). Expression of *PPARGC1A* tended to be lower in LBW-ALA piglets compared with NBW-ALA animals at 26 dpn (*p* = 0.080, Fig. 3f), although it was not altered by supplementation or age (*p* > 0.1).

### Effects of supplementation on proliferation of cultured myogenic cells

**Figure 4:**
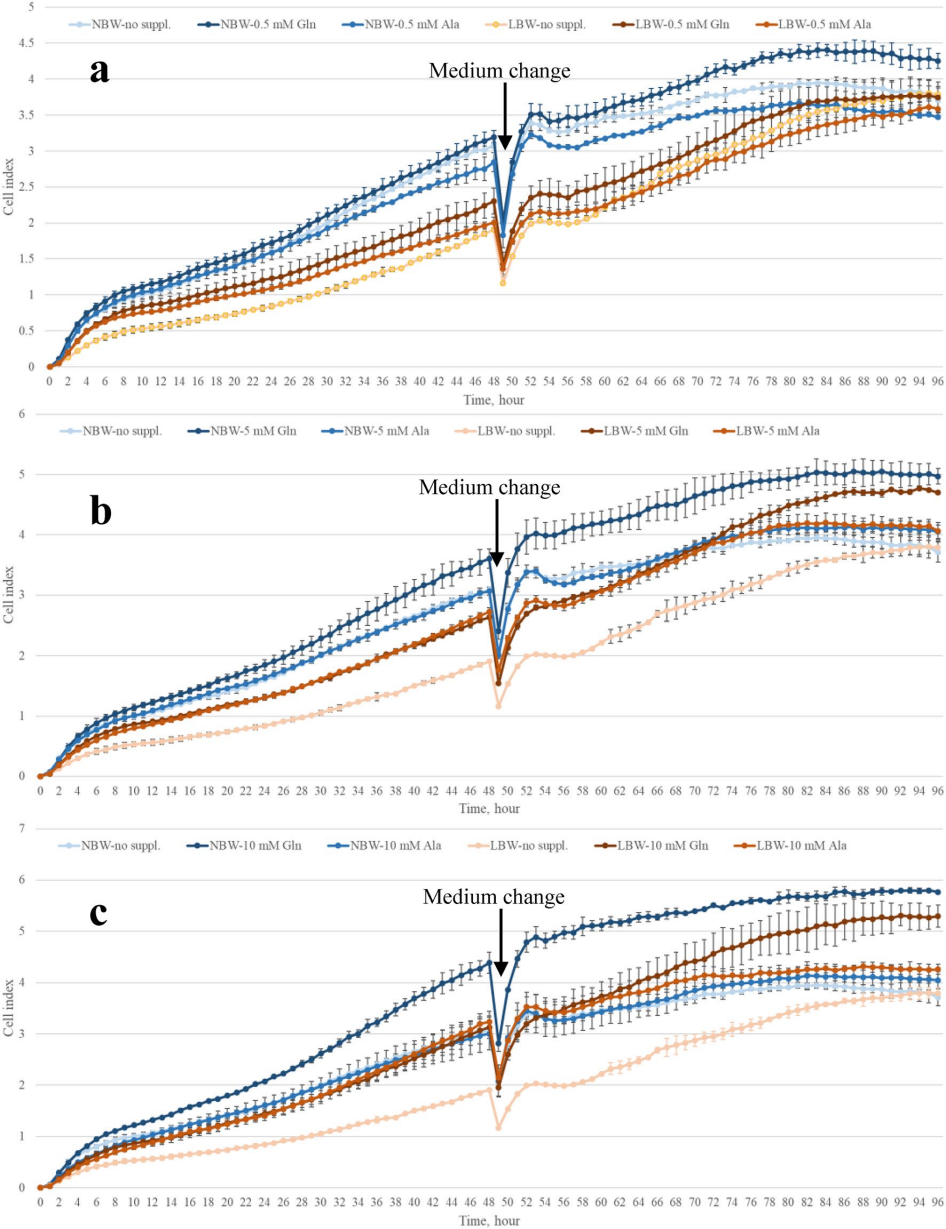
Cell indexes of proliferating myogenic cells. Cell indexes were recorded for myogenic cells with the xCELLigence RTCA-DP device. Cells, isolated from *M. longissimus* of low birth weight (LBW) and normal birth weight (NBW) piglets at 4 dpn, were supplemented with 0.5 mM Gln or Ala (**a**), 5 mM Gln or Ala (**b**), 10 mM Gln or Ala (**c**) and compared with myogenic cells without supplementation (no suppl.). The basic medium contained 0.28 mM Gln and Ala. All values are presented as Means and SD (standard deviations) of three repetitive wells.

A highly pure and vital population of satellite cells can be obtained with the established protocol developed in our lab^35, 36^. Those cells were used to conduct three independent xCELLigence assays, which demonstrated a high repeatability of the cell growth measurements. Cell indexes (CI) were continuously recorded every 15 min for each treatment over 96 h as exemplarily shown for one experiment with intervals of 1 h in Fig. 4. Low concentrations of supplements had no clear effect on recorded CI of myogenic cells. In NBW myogenic cells, CI curves were higher in cells with Gln supplementation (5 and 10 mM) compared with corresponding cells with alanine (Ala, 5 and 10 mM) or no supplementation. Of note, the basic medium (control) contained 0.28 mM Gln and Ala to ensure normal cell growth. Nevertheless, this condition was referred to as “0” (no supplementation) in the following tables and figures. Cells from LBW piglets had lower CI under the same supplementation than the cells from NBW piglets, but could catch up growth with NBW cells at final time points when supplemented with Gln at 5 or 10 mM. The CI curve of Ala (5 and 10 mM) supplemented LBW cells plateaued earlier than that of corresponding Gln supplemented cells.

Maximal cell indexes (CI_Max_) in xCELLigence assays were reached by NBW cells about 7 h earlier then by LBW cells (*p* = 0.068, Table 1). The CI_8–48 h_ (mean CI between 8 and 48 h) and the CI_56–78 h_ (mean CI between 56 and 78 h) were calculated as measures for the proliferation of myogenic cells before and after medium change, respectively. Independent of supplementation, CI_8–48 h_ or CI_56–78 h_ of LBW cells was lower or tended to be lower compared with that of NBW cells at concentrations of 0 and 0.5 mM supplementation (Table 1). The CI_56–78 h_ of 10 mM Gln supplemented cells was greater than that of 10 mM Ala supplemented cells (*p* = 0.038). Stimulation effects of different supplementations on myogenic cells were normalized to corresponding cells without supplementation from 8 to 48 h before medium change, or from 56 to 78 h before the curves reached plateau. The stimulation effect of supplements at 5 and 10 mM tended to be greater in LBW than NBW cells (*p* = 0.057 and *p* = 0.054, respectively) before medium change. Later, Gln supplementation stimulated the CI_56–78 h_ in both cell types (Table 1). In particular in LBW cells, supplementation with 0.5, 5 and 10 mM Gln increased the CI_56–78 h_ by 3.54%, 22.46% and 42.37%, respectively. The greater stimulation effect of 10 mM compared to 0.5 mM in LBW cells (*p* = 0.006) indicates dose dependency. In comparison, supplementation with the same concentrations of Ala increased the CI_56–78 h_ by 1.98%, 15.95% and 20.83%, respectively. In NBW cells (Table 1), Gln supplementation with 0.5, 5 and 10 mM enhanced the CI_56–78 h_ by 7.53%, 7.80% and 31.91%, whereas the same concentrations of Ala affected CI_56–78 h_ by − 8.25%, − 2.80% and − 12.91%, respectively. The stimulation effect of supplements at 5 and 10 mM was greater in LBW than NBW cells (*p* = 0.028 and *p* = 0.044, respectively).

**Table 1:** Growth parameters of myogenic cells from low (LBW) or normal birth weight (NBW) piglets cultivated in medium supplemented with different concentrations of glutamine (Gln) or alanine (Ala).

Trait	Suppl. concentration (mM)	LBW		NBW		p-value		
		Gln	Ala	Gln	Ala	BiW	Suppl.	BiW * Suppl.
CI_Max_	0	3.13 ± 0.37		3.67 ± 0.37		0.147		
	0.5	3.47 ± 0.37	3.35 ± 0.37	4.05 ± 0.37	3.43 ± 0.37	0.372	0.320	0.520
	5	3.91 ± 0.37	3.70 ± 0.37	4.18 ± 0.37	3.67 ± 0.37	0.760	0.337	0.751
	10	4.30 ± 0.37	3.57 ± 0.37	5.02^a^ ± 0.37	3.23^b^ ± 0.37	0.554	0.002	0.001
Time to reach CI_Max_ (h)	0	89.89 ± 3.67		82.97 ± 3.67		0.068		
	0.5	91.81 ± 3.67	91.39 ± 3.67	88.56 ± 3.67	85.39 ± 3.67	0.216	0.628	0.585
	5	90.64 ± 3.67	88.31 ± 3.67	93.31 ± 3.67	91.56 ± 3.67	0.426	0.582	0.809
	10	95.31 ± 3.67	93.47 ± 3.67	93.14 ± 3.67	90.72 ± 3.67	0.507	0.566	0.851
CI_8–48 h_	0	1.42^B^ ± 0.23		2.00^A^ ± 0.12		0.004		
	0.5	1.51 ± 0.18	1.47 ± 0.16	2.13 ± 0.20	1.94 ± 0.16	0.004	0.524	0.035
	5	1.74 ± 0.27	1.65 ± 0.24	1.93 ± 0.19	1.93 ± 0.09	0.265	0.820	0.673
	10	1.87 ± 0.37	1.76 ± 0.26	2.19 ± 0.24	1.68 ± 0.11	0.643	0.254	0.317
Stimulation effect (%, 8–48 h)	0.5	11.22 ± 14.57	7.70 ± 9.18	6.54 ± 3.55	− 2.22 ± 3.92	0.426	0.502	0.363
	5	24.33 ± 14.70	17.98 ± 16.88	− 3.22 ± 9.24	− 2.98 ± 1.97	0.057	0.804	0.212
	10	32.17 ± 18.91	25.71 ± 21.50	7.83 ± 11.81	− 14.21 ± 6.54	0.054	0.377	0.050
CI_56–78 h_	0	2.80^B^ ± 0.36		3.45^A^ ± 0.23		0.039		
	0.5	2.89 ± 0.37	2.83 ± 0.28	3.72 ± 0.32	3.17 ± 0.29	0.072	0.340	0.196
	5	3.43 ± 0.48	3.25 ± 0.51	3.73 ± 0.41	3.36 ± 0.25	0.640	0.528	0.868
	10	3.98 ± 0.57	3.36 ± 0.53	4.55 ± 0.53	2.99 ± 0.35	0.843	0.038	0.100
Stimulation effect (%, 56–78 h)	0.5	3.54^y^ ± 3.07	1.98 ± 4.22	7.53^a^ ± 2.35	− 8.25^b^ ± 2.24	0.320	< 0.001	< 0.001
	5	22.46^xy^ ± 6.90	15.95 ± 9.11	7.80 ± 8.37	− 2.80 ± 1.67	0.028	0.242	0.004
	10	42.37^x^ ± 9.20	20.83 ± 14.36	31.19^a^ ± 8.50	− 12.91^b^ ± 9.19	0.044	0.005	0.002

Values are calculated from CI recorded with the xCELLigence RTCA-DP device and are presented as LSMeans ± SE (n = 3 assays per group). Different uppercase letters (A, B) indicate significant differences between LBW and NBW cells; lowercase letters (a, b) indicate significant differences between supplementation groups within the same cell type; different lowercase letters (x, y) indicate significant differences among supplementation concentrations within the same group (*p* ≤ 0.05, Tukey–Kramer test).

*CI*_*Max*_ maximum cell index, *CI*_*8–48 h*_ mean cell index between 8 and 48 h, *CI*_*56–78 h*_ mean cell index between 56 and 78 h, *Suppl.* supplemention.

### Expression of muscle growth-related genes in proliferating myogenic cells

**Figure 5:**
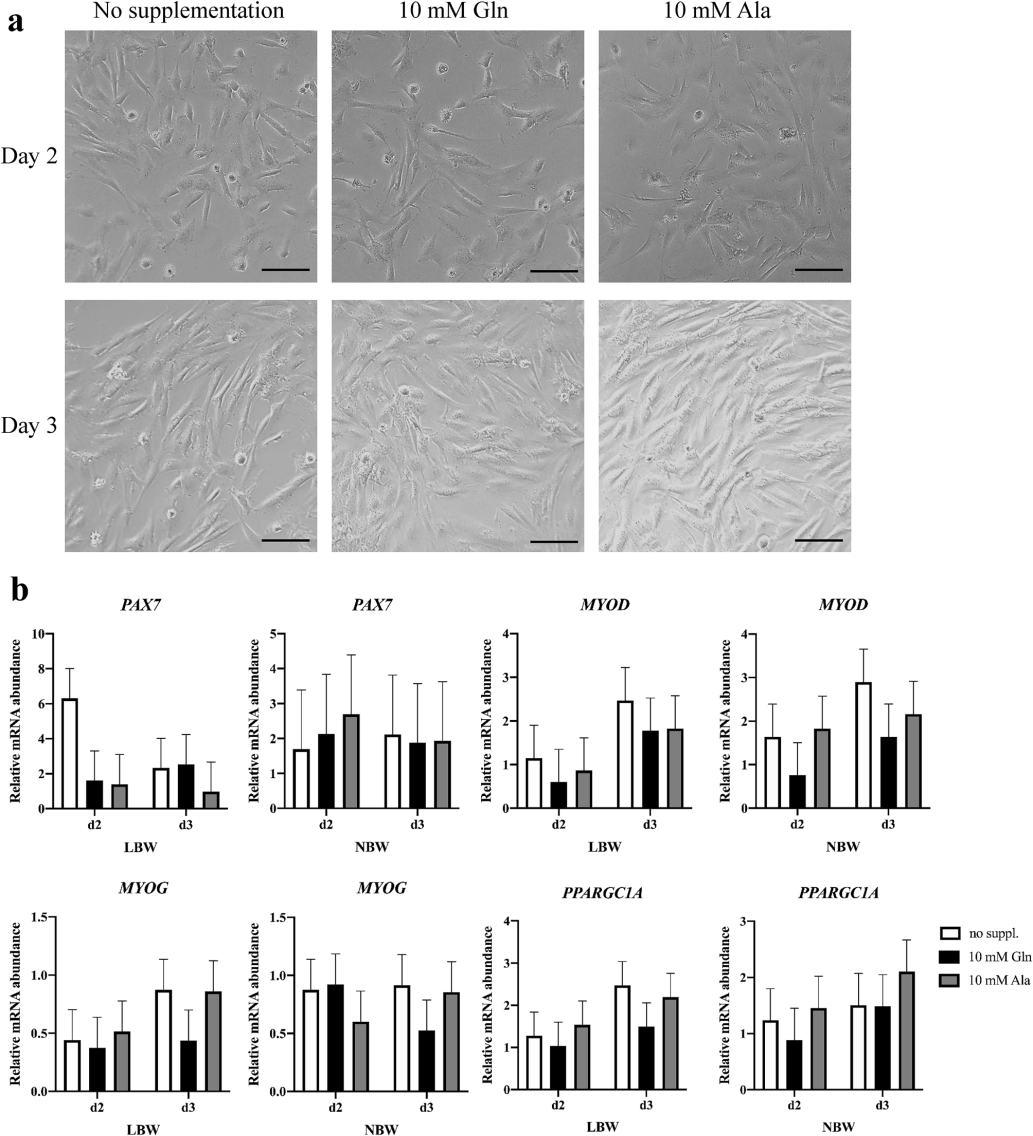
Expression of muscle growth-related genes in proliferating porcine myogenic cells. (**a**) Representative microscopic images of cells of the same sample at day 2 and day 3 without supplementation or with 10 mM Gln or Ala. Scale bars represent 200 µm. (**b**) Relative mRNA abundances of *PAX7*, *MYOD*, *MYOG* and *PPARGC1A* in proliferating cells of low (LBW) or normal birth weight (NBW) piglets at day 2 and day 3 after seeding. Values were normalized to *YHWAZ* and *PPIA* expression and are presented as LSmeans and SE (n = 3 per group).

To further investigate the effect of supplementation on the transcriptional level of muscle growth-related genes in proliferating cells, LBW or NBW myogenic cells were cultivated in growth medium supplemented with 0 or 10 mM Gln or Ala (Fig. 5a). The supplementation of 10 mM was chosen according to the xCELLigence assay results, where 10 mM Gln stimulated the CI to the highest degree. The mRNA abundances (Fig. 5b) of *PAX7*, *MYOD*, *MYOG* and *PPARGC1A* were quantified in proliferating LBW or NBW cells at day 2 and day 3 after seeding. However, no supplementation effects were observed on the mRNA level of these potential growth-related genes (*p* > 0.1).

### Expression of muscle growth-related genes in differentiating myogenic cells

**Figure 6:**
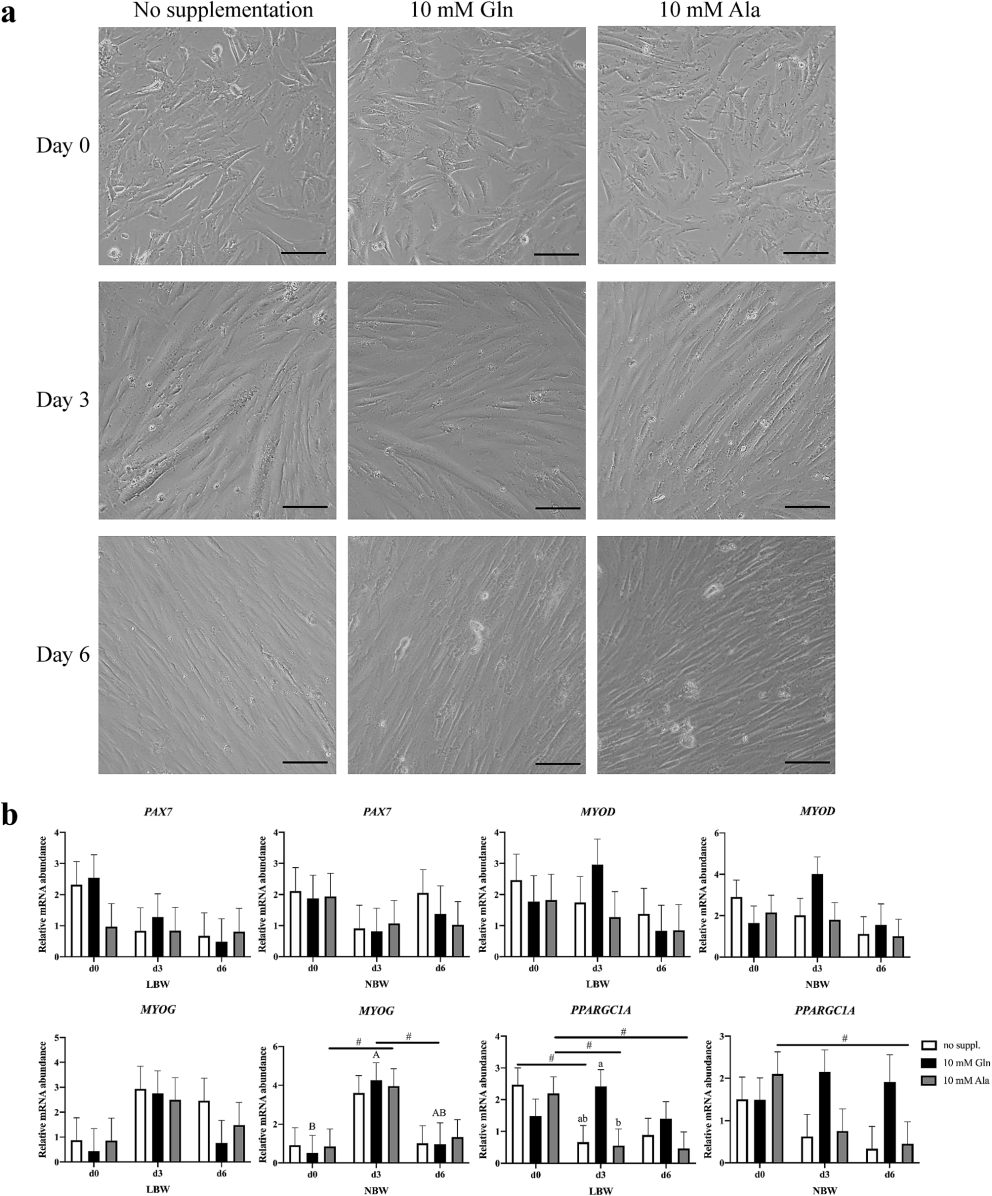
Expression of muscle growth-related genes in differentiating porcine myogenic cells. (**a**) Representative microscopic images of cells of the same sample at day 0 (day of differentiation induction), day 3 and day 6 without supplementation or with 10 mM Gln or Ala. Scale bars represent 200 µm. (**b**) Relative mRNA abundances of *PAX7*, *MYOD*, *MYOG* and *PPARGC1A* in myogenic cells from low (LBW) or normal birth weight (NBW) piglets at day 0, day 3 and day 6 after induction of differentiation. Values were normalized to *YHWAZ* and *PPIA* expression and are presented as LSmeans and SE (n = 3 per group). Different lowercase letters indicate significant differences among groups within the same time point, different uppercase letters indicate significant differences (*p* ≤ 0.05, Tukey–Kramer test) and ^#^trend for differences (0.5 < *p* ≤ 0.1) among different time points.

Myogenic cells were induced to differentiate after reaching confluence and were harvested at day 0 (day of differentiation induction), day 3 or day 6 after induction (Fig. 6a). Relative mRNA abundances of *PAX7*, *MYOD*, *MYOG* and *PPARGC1A* were determined in differentiating LBW and NBW cells (Fig. 6b). The results indicated no influence of supplementation on relative mRNA abundances of *PAX7*, *MYOD* and *MYOG* at all three time points, neither in LBW nor in NBW myogenic cells (*p* > 0.1). Expression of *MYOG* in NBW cells tended to be higher (*p* = 0.052) at day 3 in comparison with day 0 with 10 mM Ala supplementation and was higher (*p* = 0.016) with 10 mM Gln supplementation. In differentiating LBW cells at day 3, relative mRNA abundance of *PPARGC1A* was higher in 10 mM Gln supplemented cells than that in 10 mM Ala supplemented cells (*p* = 0.044). This supplementation effect was not found in NBW differentiating myogenic cells (*p* = 0.160). Furthermore, *PPARGC1A* abundance tended to decrease from day 0 to day 6 in both LBW and NBW cells supplemented with 10 mM Ala (*p* = 0.065 and *p* = 0.081), and tended to decrease in LBW cells from day 0 to day 3 with 10 mM Ala (*p* = 0.085) or no supplementation (*p* = 0.051).

## Discussion

The current study aimed at clarifying whether oral Gln supplementation affected skeletal muscle growth in LBW and NBW piglets during the early postnatal period. Glutamine supplementation could improve muscle growth in two different ways, either through direct stimulation of muscle satellite cell differentiation and fusion with muscle fibers or indirectly through enhanced nutrient accretion and supply because of improved intestinal function. The current study focused on direct effects of Gln on skeletal muscle. In our previous study, we observed an influence of Gln supplementation on muscle fiber size in MLD of these piglets^16^. If the increase in muscle fiber size was caused by a stimulatory effect on myogenic precursor cells, this could further improve the muscle fiber growth in the long term. Therefore, the current investigation was focused on the muscle cell development in vivo and in vitro and involved muscle growth-related genes. Our study indicates that LBW piglets supplemented with Gln had more total and BrdU-positive nuclei compared with Ala supplemented animals at 5 dpn, but not at 12 or 26 dpn. However, this effect was not observed in NBW piglets. It could be speculated that compared with NBW piglets, LBW piglets might use more Gln to compensate the growth delay by increased cell proliferation and/or differentiation of precursor cells. Beside myogenic precursor cells, preadipocytes may also be stimulated leading to enhanced fat deposition in LBW animals later in life. In our previous study, we observed that the total muscle nuclei number decreased with age, whereas the muscle fiber size increased^16^. In the current study, we observed a stronger reduction of the number of proliferating cells in older piglets as indicated by much less BrdU-positive nuclei. Accordingly, studies of Cardasis and Cooper^37^ and Mesires et al.^24^ reported that the number of satellite cell nuclei is reduced with age in relation to the total nuclei number. Thus, stimulation of satellite cell proliferation could be more effective in younger animals. However, skeletal muscle cells represent a heterogeneous population of cells within muscle tissue, beside muscle fibers and satellite cells there are fibroblasts, preadipocytes, adipocytes, endothelial and immune cells, etc. Fully differentiated cells, such as adipocytes and muscle fibers, are no longer able to proliferate in contrast to precursor cells^38^. Muscle fibers occupy an increasing part of the muscle cross sectional area during growth leading to a decreasing number of nuclei per area unit^39^. Our previous study has shown that intramuscular Gln availability was only increased in piglets at 5 dpn upon supplementation in NBW piglets, but not in LBW piglets^16^.

Gene expression of *PAX7*, *MYOD*, *MYF5*, *MYOG*, *PPARGC1A* and *MSTN* was quantified in MLD to elucidate the effect of Gln supplementation on these regulators of muscle growth. The role of these genes in the process of muscle development is well established, as reviewed by Zammit et al.^29, 40^, Yin et al.^41^, Schmidt, et al.^23^, Aiello et al.^42^ and Liu et al.^43^. The results of our study indicated that BiW or supplementation did not influence mRNA abundance of *PAX7*, *MYOD*, *MYF5* and *MSTN*. The expression of *PAX7* suggests that the quiescent or activated satellite cells were not altered upon supplementation or BiW differences, because *PAX7* was reported to be expressed in quiescent satellite cells, but it was co-expressed with *MYOD* when they were activated^23, 41^. Furthermore, *MYF5* and *MYOD* are involved in satellite cell proliferation and differentiation^41^, and loss of the two genes led to failure of muscle regeneration^44^. Expression of both genes was not altered in the current study. This is consistent with the results of BrdU analysis indicating that the number of total nuclei and BrdU-positive nuclei in regions exclusively filled with muscle fibers was not altered by BiW, supplementation or age. In accordance with muscle growth, transcription levels of *MYOG* and *MSTN* were higher with greater protein deposition within MLD in piglets at 26 dpn than at 5 dpn^3^. Besides, the tendency of increased *MYOG* mRNA in NBW-GLN compared with LBW-GLN piglets at 26 dpn suggests that activated satellite cells from NBW piglets had more potential to differentiate and fuse with myofibers compared with those in LBW animals, but whether the effect was regulated by Gln supplementation could not be determined. Piglets of the NBW-ALA group tended to have more *PPARGC1A* mRNA in comparison with LBW-ALA counterparts at 26 dpn, suggesting that there might be more oxidative, slow muscle fibers formed in NBW-ALA piglets^45^. Expression data of *PPARGC1A* point to the same direction like MYH7 protein abundances in the same animals reported by Zhao et al.^16^. Higher protein abundances of MYH7, representing slow, oxidative or type I fibers, were observed in NBW-ALA compared with LBW-ALA piglets at 26 dpn within MST^16^. Furthermore, *PPARGC1A* is vital for mitochondria content^46^, indicating LBW piglets supplemented with Ala but not Gln tended to have lower oxidative capacity within skeletal muscle compared with their NBW littermates. A recent study reported that *PAX7*, *MYOD*, *MYF5* and *MYOG* within *M. semitendinosus* were downregulated in female piglets suffering from intrauterine growth retardation (IUGR) compared with normal pigs from birth to 100 dpn^47^. The discrepancy with our study might result from gender, developmental stage of pigs, and the BiW definition of LBW pigs.

To verify the effects of different concentrations of Gln in cultured myogenic cells from skeletal muscle, an in vitro model was applied using cell pools generated from MLD of 2 LBW or 2 NBW piglets at 4 dpn, respectively. Cell pooling helped to ameliorate the biological variance among animals and was necessary to overcome the shortage of isolated satellite cells from single animals^48^. The proliferation capacity of cultured myogenic cells was investigated in three independent experiments using the xCELLigence device. Continuous real-time monitored CI reveal the adhesion and proliferation of the cells^49^. The results indicated a general effect of BiW independent of supplementation, indicating that the myogenic cells from LBW piglets had lower proliferation capacity. Nissen et al.^50^ likewise observed a lower number of viable cells after 3 days of cultivation of LBW porcine satellite cells isolated from *M. semimembranosus* compared with cells from normal or high birth weight animals. Additionally, fewer myogenic cells could be isolated per gram MLD from 4-day-old LBW piglets than from muscle of their NBW littermates^51^. The lower number and delayed growth of LBW myogenic cells might be the reason of retarded body growth and prolonged finishing time of LBW pigs^2^. Regarding the concentration effects of Gln supplementation on cultured muscle cells, Wu and colleagues reported a positive effect of Gln concentration applied in cultured chicken skeletal muscle and 15 mM Gln stimulated protein synthesis in cultured muscle cells by 58% or 19% in the presence or absence of tyrosine, respectively^52^. In line with their results, cultured myogenic cells from both NBW and LBW piglets, in the current study, exhibited the highest stimulation effects from 56 to 78 h with 10 mM Gln supplementation compared with 10 mM Ala, in the presence of 36 mg/L tyrosine. This confirms the positive dosage-dependent effects of Gln in cultured porcine primary muscle cells.

Since 10 mM Gln was most effective in promoting proliferation of cells in the xCELLigence assays, we applied the same supplementary concentration of Gln to the satellite cells during proliferation and differentiation in comparison to cells cultivated without supplementation or with 10 mM Ala. Expression of myogenic regulatory factors (*MYOD*, *MYOG*) as well as *PAX7* and *PPARGC1A* was quantified in the proliferating and differentiating myogenic cells as indicators for growth. However, no significant effect of supplementation was observed in the expression of those genes during the proliferation period. This suggests that these regulators were not involved in the effects of Gln supplementation on proliferating cells observed in xCELLigence assays. Moreover, expression of *MYOG* in NBW cells supplemented with 10 mM Gln increased from day 0 to day 3 after induction of differentiation and then tended to decrease from day 3 to day 6, indicating more cells in terminal differentiation at day 3 after induction^41^. Similarly, there was a trend for higher *MYOG* mRNA abundance at day 3 compared with day 0 of differentiation induction in NBW cells with Ala supplementation. However, this effect was not observed in LBW cells. Thus, this suggests that Gln has only minor positive effects on cell differentiation. Fewer LBW cells were differentiated compared with NBW cells under the same conditions, indicating the general developmental delay of these cells that could partly explain the retarded growth of LBW piglets in concordance with Rehfeldt et al.^2^. Expression of *PPARGC1A* tended to decrease from day 0 to day 6 in LBW and NBW cells with 10 mM Ala and from day 0 to day 3 in LBW cells without supplementation, suggesting a trend for decreasing oxidative activities in cells without Gln supplementation according to Rowe et al.^46^. Notably, in differentiating myogenic cells at day 3 after induction, *PPARGC1A* expression was higher in LBW myogenic cells supplemented with 10 mM Gln compared with 10 mM Ala, indicating that Gln supplementation supported higher oxidative activities^45, 46^. This is consistent with the in vivo analysis of *PPARGC1A* expression in the current study.

We observed a dose dependent stimulation effect of Gln supplementation in xCELLigence assays with the largest effect at 10 mM Gln. However, this is much higher than the physiological concentration of plasma Gln in pigs (~ 0.5 mM) that could be increased by oral administration of 0.5 g/kg BW twice daily to ~ 0.7 mM^53^. Thus, we applied a supplemental concentration of 0.5 mM Gln (0.78 mM final concentration) in the cell culture similar to the reported plasma concentration after supplementation^53^. This Gln concentration, however, induced no clear effects. Given the fact, that even this concentration may not be maintained in plasma of piglets after supplementation once daily, it might explain why the Gln supplementation to the animals had only minor effects in promoting skeletal muscle development in vivo^16^. The intramuscular concentration of free Gln was determined between 1 and 9 mmol/kg fresh matter for piglets in our study^16^, with highest values upon Gln supplementation. The Gln concentration may be high enough to stimulate cell proliferation in muscle tissue in a short term, but it is not feasible to keep the intramuscular Gln concentration at such a high level in a live animal in a long term. This may be due to the short half-life of Gln in blood (0.65–0.7 h according to Wu et al.^54^). Consequently, the elevated intramuscular Gln concentration upon supplementation was only observed in piglets at 5 dpn, indicating that the oral supplementation might have only minor modulating effects for the intramuscular Gln concentration and thus for the stimulation of satellite cells. Furthermore, cultured satellite cells function in a different way as those in vivo because they lack regulation by niche environment including regulating niche factors and complex signaling pathways^41, 55^. Thus, the high proliferation possibility of satellite cells might be restricted to in vitro conditions*,* although supplementation in vivo was applied during the early postnatal period of piglets. Our study indicated that LBW myogenic cells caught up growth upon Gln supplementation to the level of NBW cells in the xCELLigence assays, but this could not be confirmed in vivo in LBW compared to NBW piglets.

Taken together, Gln supplementation stimulated proliferation of different, undefined cell types within MLD of early postnatal piglets. However, the mRNA abundances of muscle growth-related genes were not affected by Gln supplementation and were only slightly influenced by BiW. Glutamine supplementation promoted proliferation of cultured myogenic cells, isolated from *M. longissimus* of LBW and NBW piglets in a dose dependent manner. In conclusion, the oral Gln supplementation has some potential to improve muscle cell development, but the positive effects of applicable doses are not clear enough to justify the higher effort.

## Methods

All experimental procedures and animal care were carried out in agreement with the instructions of European Convention for the Protection of Vertebrate Animals used for Experimental and Other Scientific Purposes (2010/63/EU) and were approved by the ethics committee of the State Office for Agriculture, Food Safety and Fisheries Mecklenburg-Western Pomerania, Germany (permission No. 7221.3-1-026/16). The study was carried out in compliance with the ARRIVE guidelines^56^.

### Animals and sampling

The current study comprised 144 male German Landrace piglets, littermates with low or normal birth weight as described recently^16^. The piglets were assigned to four groups as LBW (n = 72) or NBW (n = 72) piglets, supplemented with Gln (1 g/kg body weight) or an isonitrogeneous amount (1.22 g/kg body weight) of Ala (group names: LBW-GLN, LBW-ALA, NBW-GLN and NBW-ALA). Twelve piglets per group were stunned by captive bolt and exsanguinated at 5, 12 and 26 dpn. One hour before slaughter, the piglets were injected intraperitoneally with BrdU (12 mg/kg body weight, Merck, Darmstadt, Germany). Tissue of MLD was sampled after slaughter from the left side of the carcass, cut into small pieces, snap frozen in liquid nitrogen, and stored in − 80 °C until further analysis.

Additionally, untreated male and female German Landrace piglets were used to isolate myogenic progenitor cells from MLD for primary cell culture as described below. Cells from 6 piglets with LBW (0.92 ± 0.03 kg) and their respective NBW littermates (1.38 ± 0.03 kg) at 4 or 5 dpn were used for proliferation assays with xCELLigence (RTCA-DP, ACEA Biosciences, San Diego, CA, USA) and for cell proliferation and differentiation assays.

### Immunohistochemistry

Muscle sections from MLD were cut 8 µm thick with a cryostat microtome (CM3050 S, Leica, Bensheim, Germany). The sections were fixed in 4% paraformaldehyde solution for 20 min, washed 2 × 5 min with PBS (phosphate-buffered saline) and permeabilized with 0.1% TritonX-100 (Sigma-Aldrich, Munich, Germany) in PBS for 10 min. Then, the slides were incubated with 2 N HCl at 37 °C for 60 min to denature DNA. After washing 3 × 5 min with PBST, nonspecific bindings of the secondary antibody were blocked with 10% normal goat serum (NGS) in PBST for 15 min at room temperature. The slides were incubated overnight at 4 °C with the primary mouse anti-BrdU antibody (1:100 in PBST incl. 1% NGS) in a humidity chamber. After washing 3 × 10 min with PBST, slides were incubated with the secondary antibody (Alexa Fluor 488 goat anti-mouse IgG, 1:1000 in PBST, Thermo Fisher Scientific, Schwerte, Germany) for 45 min at room temperature in the dark and then washed 3 × 5 min with PBST. Nuclei were counterstained with propidium iodide (PI, 5 µg/mL, Sigma-Aldrich) in the dark for 10 min. Finally, slides were washed 1 × 5 min with PBS and 1 × 10 min with distilled water and covered with ProLong Diamond Antifade Mountant (Thermo Fisher Scientific) and coverslips (Roth, Karlsruhe, Germany). Immunofluorescence of BrdU and PI stained nuclei was detected using a Nikon Microphot SA fluorescence microscope (Nikon, Duesseldorf, Germany) equipped with a CC-12 color camera (OSIS, Münster, Germany) and Cell^F image analysis software (OSIS). Two macro programs were developed to determine either the total nuclei area percentage (stained red) as well as the area percentage of BrdU-positive nuclei (stained green), or the number of muscle fiber nuclei (nuclei within muscle fibers and nuclei of satellite cells) and corresponding BrdU-positive nuclei. Eight randomly selected pictures, of approximately 2.1 mm^2^ in total, were analyzed for nuclei area percentages and five regions, of about 0.32 mm^2^ in total, for nuclei numbers from each piglet. The ratios between BrdU-labeled areas or nuclei numbers and total nuclei area or numbers, respectively, were determined as measures of intramuscular cell proliferation.

### Isolation of myogenic progenitor cells

The cell isolation protocol was adapted from Mau et al.^57^ with moderate adjustments. Briefly, muscle tissue of MLD was collected immediately after slaughter from LBW or NBW piglets and kept in the isolation medium, which consisted of 80% PBS-D (144 mM NaCl, 5.4 mM KCl, 25 mM glucose, 14 mM sucrose, 5 mM Na_2_HPO_4_, 0.5% penicillin–streptomycin (10,000 U/mL penicillin, 10 mg/mL streptomycin, PAN-Biotech, Aidenbach, Germany), and 1 mg/L phenol red, pH 7.4), 10% penicillin–streptomycin and 10% amphotericin B (250 μg/mL). Then, the MLD tissue was rinsed with PBS-M (137 mM NaCl, 2.7 mM KCl, 3.2 mM Na_2_HPO_4_, pH 7.4) with antibiotics and 70% ethanol. After removal of visible extraneous tissue, the MLD tissue was minced into pieces, as small as possible, with scissors in a petri dish with 10 mL HBSS (PAN-Biotech). The muscle pulp was digested with 100 mL trypsin solution (100 mL 4232 U/mL trypsin, Sigma-Aldrich and 5.8 ml HBSS) per 50 g muscle tissue at 37 °C for 1 h with a sterilized magnetic stirrer at 250 rpm. Then, the digestion solution was transferred to falcon tubes, equal amounts of growth medium (77.5% Alpha MEM Eagle (Sigma-Aldrich), 20% fetal bovine serum (FBS, Gibco), 1% penicillin–streptomycin, 1% amphotericin B and 0.5% gentamicin) were added and the tubes were centrifuged at 800 × *g* for 10 min. After centrifugation, the pellet without supernatant was resuspended with growth medium and filtered with a funnel and sterilized gauze together with the resuspension from the last step. The filtrate was centrifuged at 1200 × *g* and 4 °C for 15 min. Myogenic precursor cells were isolated with percoll (GE Healthcare Life Sciences, Freiburg, Germany) density gradient centrifugation (100%, 70% and 40%). Cells at interface between 40 and 70% of the gradient layers were recovered, diluted with growth medium in a new tube and centrifuged. The pellet was resuspended with growth medium and the cell number was counted with an automated cell counter (Thermo Fisher Scientific). Previous studies demonstrated that the vast majority of cells isolated with this method are satellite cells that are able to differentiate. Abundance of myogenic markers were demonstrated with flow cytometry and the ability to terminally differentiate and generate myotubes was shown^35, 36^. For xCELLigence assays (see below), myogenic cells from single animals were seeded in 10 cm collagen-coated culture dishes (Greiner Bio-One, Kremsmünster, Germany) and incubated at 37 °C, 6% CO_2_ and 95.5% humidity. For proliferation and differentiation assays, cells from the same birthweight (BiW) group (4 donors per group) were pooled after isolation and cultivated under the same condition as aforementioned. Growth medium was replaced after 24 h. After 4 days, cells were collected using trypsin/EDTA solution in PBS (0.05%/0.02%, Biochrom, Berlin, Germany) and mixed with equal amounts of freezing solution containing 50% culture medium, 20% dimethyl sulfoxide (DMSO, SERVA, Heidelberg, Germany) and 30% FBS and stored in cryo tubes. The cells were immediately stored at − 80 °C for 24 h and then transferred to liquid nitrogen for long-term storage. Frozen cells were used for the subsequent cell culture experiments.

### Monitoring of growth kinetics with xCELLigence

Growth medium without Gln including 77.5% Gln-free Alpha MEM Eagle (Sigma-Aldrich), 20% FBS, 1% penicillin–streptomycin, 1% amphotericin B and 0.5% gentamicin, was prepared for different concentrations of Gln and Ala growth medium. To encounter the same concentration of 0.28 mM Ala in the basic commercial Alpha MEM Eagle medium, Gln concentration was adjusted to 0.28 mM with 200 mM liquid Gln solution (Sigma-Aldrich) as well. Thus, the basic medium with no supplementation of Gln could still supply the cells with a certain small amount of Gln to sustain the cell growth and mimic the physiological environment of muscle cells. The different supplementation concentrations (0.5, 5, 10 mM) of Gln or Ala (Sigma Aldrich) were added to the original medium with 0.28 mM Gln and Ala. Frozen LBW or NBW myogenic cell suspensions were quickly thawed in a 37 °C water bath. Then, the cells from two donors with similar BiW were pooled and seeded in 10 cm collagen-coated culture dishes with growth medium, without Gln supplementation. After 3 days, the cells were harvested with trypsin/EDTA solution in PBS and prepared for xCELLigence experiments. In brief, 100 µl warm growth medium with different concentrations of Gln or Ala (0, 1, 10 and 20 mM, which was diluted later to 0, 0.5, 5 and 10 mM with resuspended cells) was added to the xCELLigence E-plate (OMNI Life Science, Bremen, Germany), and the E-plate was placed on the xCELLigence RTCA-DP instrument. The background data of the medium without cells was measured with a RTCA Software 2.0 (ACEA Biosciences). Then, the cells resuspended with 100 µl growth medium without Gln were seeded into the wells (1 × 10^4^ cells per well) of the xCELLigence E-plate and monitored with the RTCA software under the same condition as cultivation after cell isolation. Each supplementation was performed in triplicates in three independent xCELLigence assays. The whole xCELLigence assay was continuously monitored and the CI was recorded every 15 min over a time period of 96 h. The growth medium with different concentrations of supplementation was changed after 2 days.

### Cell proliferation and differentiation assays

Myogenic cells were cultivated and harvested at different time points during proliferation and after induction of differentiation to determine gene expression of myogenic genes. Cell pools from LBW and NBW piglets, respectively, were thawed and seeded on Matrigel (Corning, New York, USA) coated 24-well plates (Sarstedt, Nümbrecht, Germany) with 5 × 10^4^ cells in each well. For proliferation, cells were cultivated with 0 mM Gln, 10 mM Gln or 10 mM Ala supplemented growth medium. Cells were collected with Qiazol lysis reagent (Qiagen, Hilden, Germany) at day 2 and 3 after seeding. For differentiation, cells were cultivated in the same way during the proliferation period until day 3. At day 3 (80–90% confluence of cells), differentiation medium (96% Gln-free Alpha MEM medium, 2% FBS, 1% penicillin–streptomycin, 1% amphotericin B and 0.28 mM Gln) supplemented with 0 mM Gln, 10 mM Gln or 10 mM Ala was applied to the cells after washing once with PBS. The differentiation medium was replaced every 3 days. The myogenic cells were collected with Qiazol lysis reagent at days 0, 3 and 6 after induction of differentiation. For each plate, duplicate wells of supplementation were performed in three independent cultivation experiments.

### RNA isolation and cDNA synthesis

Muscle RNA was extracted with an RNeasy Fibrous Tissue Mini Kit (Qiagen) from 70 to 90 mg of MLD, while RNA of cultured myogenic cells was isolated with Qiazol lysis reagent following the standard protocol. All RNA was stored in − 80 °C until subsequent analysis. A NanoDrop ND-1000 spectrophotometer (Peqlab, Erlangen, Germany) was used to determine RNA concentration; and RNA integrity was determined using the Experion Automated Electrophoresis System and the RNA StdSens analysis chip (Bio-Rad, Munich, Germany) following the manufacturer’s instructions. Then, cDNA was synthesized with an iScript cDNA synthesis kit (Bio-Rad) in a 20 μL reaction volume from 1000 ng (muscle tissue) or 600 ng RNA (myogenic cells) and stored in − 20 °C. Primers of reference and target genes were adopted from literature^58–63^, shown in Table 2. All primers were synthesized by a commercial company (Sigma-Aldrich). The annealing temperature of all primers was 60 °C. Qualitative polymerase chain reaction (PCR) was performed and the products were subjected to agarose gel electrophoresis to test the primers and products.

**Table 2:** Primer sequences for qPCR.

Gene	Accession number	Forward primer (5′–3′)	Reverse primer (5′–3′)	Size (bp)
*YWHAZ*^58^	NM_001315726.1	ATGCAACCAACACATCCTATC	GCATTATTAGCGTGCTGTCTT	178
*PPIA*^59^	NM_214353.1	CACAAACGGTTCCCAGTTTT	TGTCCACAGTCAGCAATGGT	171
*PAX7*^60^	AY653213.1	CAACCACATCCGCCACAA	TCTTGGAGACACAGCCATGG	101
*MYF5*^61^	NM_001278775.1	CCTGAATGCAACAGCCCT	CGGAGTTGCTGATCCGAT	152
*MYOD*^61^	NM_001002824.1	GGTGACTCAGACGCATCCA	ATAGGTGCCGTCGTAGCAGT	108
*MYOG*^61^	NM_001012406	CAACCAGGAGGAGCGAGAC	AGGGTCAGCTGTGAGCAGAT	161
*MSTN*^62^	NM_214435.2	CCCGTCAAGACTCCTACAACA	CACATCAATGCTCTGCCAA	141
*PPARGC1A*^63^	NM_213963	CCCGAAACAGTAGCAGAGACAAG	CTGGGGTCAGAGGAAGAGATAAAG	111

### Quantitative PCR (qPCR)

The qPCR was performed with FastStart Essential DNA Green Master using a LightCycler 96 real-time qPCR system (Roche, Basel, Switzerland) as described elsewhere^64^. Quantitation cycle (Cq) value was calculated by the LightCycler 96 system software. All samples were measured in duplicates. Efficiencies of amplifications were analyzed with standard curves from qPCR with serial cDNA dilutions (1, 1/10, 1/50, 1/100, 1/200) and were within 1.8–2.1. The mRNA abundance of target genes was normalized to two reference genes, tyrosine 3-monooxygenase/tryptophan 5-monooxygenase activation protein zeta (*YWHAZ*)^58^ and peptidylprolyl isomerase A (*PPIA*)^59^, and was calculated as normalized relative quantities (NRQ)^65^.

### Statistical analysis

Data were subjected to analysis of variance (ANOVA) with the MIXED procedure of SAS statistical software (Version 9.4, SAS Inst., Cary, USA). For animal data analyses, BiW (LBW, NBW), supplementation (Gln, Ala), age (5, 12, 26 dpn) and their respective interactions were included as fixed factors and sow as random factor. The SLICE statement was used to enable the partitioned analysis of the least-squares means (LSmeans) for the interaction between BiW and supplementation within the same age. For analysis of xCELLigence assays, BiW (LBW, NBW), supplementation (Gln, Ala), concentration of supplementation (0, 0.5, 5, 10 mM), and their respective interactions were included as fixed factors. Cell indexes in time intervals before (8–48 h) and after (56–78 h) medium change as well as the respective stimulation effects were analyzed using the same model, but as repeated measurements with “compound symmetry” as covariance structure and group effect BiW × supplementation × concentration. For analysis of myogenic cell proliferation and differentiation assays, BiW (LBW, NBW), supplementation (no supplementation, Gln, Ala), day of proliferation (2, 3) or differentiation (0, 3, 6) and their respective interactions were considered as fixed factors. The SLICE statement of the MIXED procedure was used to enable the partitioned analysis of the LSmeans for the interaction between supplementation within the same cultivation day. Tukey–Kramer test was applied to analyze pairwise differences. Values are presented as LSmeans and standard errors (SE). Differences were considered significant if *p* ≤ 0.05, or a trend if 0.05 < *p* ≤ 0.1.
